# Digital Transformation and Financial Risk Prediction of Listed Companies

**DOI:** 10.1155/2022/7211033

**Published:** 2022-09-12

**Authors:** Chen Xinxian, Cai Jianhui

**Affiliations:** ^1^School of Accounting, Guangdong University of Finance, Guangzhou, Guangdong 510521, China; ^2^School of Tourism Management, Sun Yat-Sen University, Guangzhou, Guangdong 510275, China

## Abstract

Digitalization is a revolution, a frontal battleground in the new global competitive landscape, and a long-distance race for which all employees must be prepared, and organizations must actively embrace the resulting changes. The article begins by analyzing three characteristics of digital transformation and enterprise growth: the heterogeneity of digital transformation's impact on enterprise growth and the process by which digital transformation influences enterprise growth. In addition, this article develops a convolutional neural network-based financial early warning model to aid businesses' digital transformation initiatives.

## 1. Introduction

Deep integration of new generation digital technology with the real economy has aided the transition of global economic development momentum from the traditional economy to the digital economy, thereby accelerating the transition from the traditional economy. In terms of importance, a new economic and social development engine has emerged, joining land, capital, and labor. Currently, the rising cost of production inputs, an aging population, and resource and environmental constraints are impediments to China's economic restructuring and industrial upgrading. The digital economy is widely regarded as a viable strategy for addressing the current challenges of economic development. As is the case in numerous other nations, the Chinese government continues to pay close attention to the growth of the digital economy [1–4]. In 2017, at the 19th National Congress of the Communist Party of China, a report was issued that forecasted the future development of the digital economy. General Secretary Xi Jinping stated multiple times in 2020 that in order to achieve sustainable development, it is crucial to exploit the potential offered by industrial digitization and digital industrialization, as well as to pay special attention to the digital architecture of the economy. There are two distinct phases of the digital economy: digital industrialization and industrial digitization, with digital transformation constituting a substantial portion of industrial digitization. Digital transformation is the process by which businesses use digital technology to reduce duplication of effort or to replace traditional digital technology with advanced digital technology in the course of manufacturing, operating, and providing customer services. Currently, domestic enterprises undergoing digital transformation face three major challenges: a reluctance to transfer because of high transformation costs, a willingness to transfer because of a long-term commitment to transformation, and a failure to transfer because of the lack of transformation capabilities [5–9].

The introduction of advanced digital technologies (such as the Internet of Things, big data computing, and artificial intelligence) into production management, organization, operation, and research and development, and innovation is referred to as the digital transformation of an enterprise. At the theoretical level, it may optimize the allocation of internal and external resources of the enterprise, increase the ability of the enterprise to achieve sustainable development, and finally achieve scale growth and efficiency improvement, while also promoting the growth of the enterprise. However, the truth is more complicated, with some researchers evaluating more than 150 studies and concluding that there is insufficient evidence to support digital investment in terms of increasing output and productivity [10, 11]. According to certain academic research, Portugal's ongoing investment in the use of digital technology has not resulted in a rise in worker productivity, and there is no statistically significant association between the two. In addition, according to the statistics from the survey of Chinese firms, just 11 percent of enterprises' digital transformation will result in genuine economic gains by 2020, and the majority of enterprises will face numerous challenges in their digital transformation efforts. Because of this, certain fundamental concerns in the field of digital transformation must be addressed as soon as possible, including what influence will digital transformation have on enterprise growth? What kind of heterogeneity does digital transformation exhibit depending on the circumstances in which it occurs? What are the specific mechanisms via which digital transformation has an impact on the growth of an enterprise? The responses to the questions will contribute to a better knowledge of the digital economy, will assist businesses in better formulating digital transformation strategies, and will serve as a reference for government departments in guiding the digital development of industries [12, 13].

It is the goal of the financial crisis early warning (FCEW) model to predict the likelihood of enterprise risk in advance by establishing a correlation between important indicator data information and financial risk in the business's financial statements. The FCEW results not only assist the firm in taking timely remedies to better financial management, but they also contribute to the healthy and steady growth of the capital market as a result of their findings. When looking at the research trends in FCEW at home and abroad, it is primarily concerned with the FCEW indicator and the FCEW model, amongst other things. The existing study on the selection of FCEW indicators is primarily concerned with determining which indicators are most accurate in predicting the crisis status of businesses. The experience begins with a single financial ratio indicator, progressing to a multivariable financial ratio indicator, then with multidimensional indicators mixed with financial indicators and nonfinancial indicators, and finally, with the common application stage. Although the studies mentioned above have yielded some forecasting results, the identification of multidimensional early warning signs is mainly accomplished through the application of statistical methods. Methods normality and significance tests were performed on the sample index data, which were then combined with manual discrimination based on the professional capacity to choose indicators for use in the study [14–19]. The selection technique was difficult, and there was no uniform conclusion regarding which indicators should be used to make the decision. Early scholars frequently used univariate models, logistic models, discriminant analysis models, and other types of models in their selection of early warning models. With the advancement of information technology, it is now possible to get a huge amount of publicly available real-time data information while doing business operations. These statistics can frequently indicate the risk status of the firm in the past, and they can even reflect some features of the risk of the entire capital market in some cases. Because of this, researchers began applying artificial intelligence approaches such as neural networks and support vector machines to the early detection of financial crises. Early warning models based on artificial intelligence approaches are, in general, included in the early warning system. Although the system has made significant strides, it still faces significant challenges, including overcoming the limitations of statistical method-based early warning models, which require data to follow a normal distribution, complex calculation, and analysis, as well as having strong fault tolerance and learning capabilities. The current artificial neural network has evolved to do the following functions: in addition to being used in the field of FCEW, the deep learning network stage is characterized by self-learning and high dynamic adaptability [20–26].

Based on a comprehensive analysis of enterprise operations, this study develops a system of early warning indicators that integrates financial and nonfinancial indicators while considering the current state of available indicators. The PCA method is then used to reduce the dimensionality of the alternative indicators' data and eliminate those that have a significant impact on financial risks. While employing a relatively simple method to filter interference information, the principal component with a major effect can contribute to enhancing the research outcomes of a large financial early warning indication selection method that employs a principal component with major impact. Second, the FCEW model, which is based on the DL theory, is presented to identify and extract information from a large volume of real-world data pertaining to the company's previous operations. Effective features and laws, as a result of the early warning model's continuous self-learning, improve the model's forecast accuracy and stability, allowing for more accurate and stable predictions of enterprise operations.

The framework of this paper is as follows: the first chapter is an introduction, which introduces the shortcomings of the previous work and the contribution of this paper; the second chapter is a relationship between digital transformation and enterprises; the third chapter is the method proposed in this paper; the fourth chapter is the experimental results; the last one is the conclusion part.

## 2. Relationship between Digital Transformation and Enterprises

Although digital transformation is not a novel concept, the digitization of government and business has become a popular topic of discussion. With the advancement of information and communication technology and the advent of the Internet, it has become increasingly popular among businesses for use in their manufacturing and operations activities. Since 1995, when Dell, IBM, and other companies began using it, the digital transformation of sales links has been in progress. These businesses use the Internet to sell terminal products directly to customers, as opposed to the conventional method of relying solely on dealer shops. The transformation of digital technology began after the year 2000. This technology is widely used in a variety of fields, including hotel room reservations, online shopping, IC bank cards, and ATMs. The development of China's industrial digital transformation can be roughly divided into three stages: the first stage, driven by ICT information and communication technology, which is characterized by the widespread use of landline telephones and fax machines in commercial organizations; the second stage, driven by ICT information and communication technology; and the third stage, driven by ICT information and communication technology. Internet-driven network transformation constitutes the second stage. Enterprises are beginning to use the Internet to conduct commercial transactions and implement manufacturing process automation technology. Lastly, technologies such as cloud computing, big data analytics, artificial intelligence, and mobile Internet access fuel the digital transformation. In the last decade, the accumulation of digital technology has produced a new generation of advanced digital technology that has begun to significantly alter the production and business activities of social organizations. In the manufacturing industry, technological advancements such as the Internet of Things and industrial Internet, as well as machine-to-machine communication, artificial intelligence, and machine vision, are being used extensively to transform traditional production methods. Digitalization has spawned new business models (such as free business models), regional blockchain distributed accounting, electronic payment, paperless workflow, and other innovations in the service industry [27–29].

### 2.1. Digital Transformation and Enterprise Growth

Enterprise expansion, according to the classical economic theory, is primarily influenced by economies of scale. The search of economies of scale is the internal driving force behind enterprise expansion, and it is this quest that serves as the internal driving force. However, enterprise expansion is a process of constant adjustment and improvement that leads to the achievement of an optimal scale in the long run. According to the new institutional economics, the expansion of companies is manifested not only in the extension of functions but also, and perhaps more importantly, in the expansion of borders. Many transaction activities that were previously handled through the market were absorbed inside the enterprise as a result of this process of boundary expansion, hence lowering overall market transaction costs. In general, the expansion of a business involves at least two aspects: the first is the enterprise's viability and the second is the expansion of the enterprise's market share. In today's strong market competition, the viability of a company is the cornerstone for its long-term development, and the viability of an enterprise is dependent on its technological capabilities. Companies with long-term viability can only benefit from high product value and innovative thinking in order to have larger development chances. The second characteristic is the ability of an organization to achieve long-term growth. Enterprises rely on their ability to differentiate themselves from the competition in order to survive in the market. Regardless of whether the organization is experiencing success or adversity, it must have the ability to surpass itself and continue to innovate in order to achieve sustainable development [30, 31].

Technical revolutions modernize and update the entire production system, which leads to new manufacturing methods. Digital technology has revolutionized product creation, service delivery, and business operations. As a term in information and communication science, digitization is the process of turning analog information into digital form and natural language into machine language. Digital technology underpins modern science and technology. Telecommunications, the Internet, the Internet of Things, robotics, machine vision, and other technologies have developed on this foundation. “Digital transformation of organizations” is the process of using digital technology to reduce duplication of effort or replace traditional digital technology with more advanced technology in manufacturing, operation, and service. Digital transformation improves operational efficiency and automation.

### 2.2. Heterogeneity

Entrepreneurs and business leaders have a crucial role in making critical decisions, such as those related to digital transformation. While state-owned firms are not simply market participants seeking profits, but they also bear the responsibility of ensuring employment and sustaining social stability in comparison to nonstate-owned enterprises. In this setting, executives of state-owned enterprises exhibit characteristics that are similar to those of quasi-officials. The executives of state-owned firms will therefore have a greater motivation to restructure their organizations when the government sets the goal of energetically promoting the digital economy as a policy objective. At the same time, under the pressure of the official promotion tournament and the target accountability system, local government officials will take the initiative to ask state-owned enterprises to bear the policy burden, that is, digital transformation and implicitly promise to provide state-owned enterprises with transformation subsidies, tax relief, or other resource preferences in exchange for bearing the policy burden. As a result, during the early stages of digital transformation, the effect of digital transformation in state-owned firms may be superior to the effect of digital transformation in nonstate-owned enterprises.

The peer effect influences a company's transformation decision in addition to its own development requirements. The current round of digital transformation is still a complex system engineering project with a high price tag and significant risk. Those businesses that initiate digital transformation will be faced with a great deal of unpredictability. In order to avoid the costs associated with trial and error, businesses typically look for a comparable reference, emulating the digital transformation initiatives of other businesses. If there is a precedent for the implementation of digital transformation among industry peers or urban peers and if organizations take the initiative to replicate and enhance the approach in the future, the impact of digital transformation on corporate growth should be significantly enhanced. Peer-to-peer competition will not only encourage enterprises to support digital transformation and strive to outperform their competitors, but it will also help them gain a competitive advantage [32–36].

Digital technology, which has its roots in the field of information and communication science, has the potential to be fully integrated with the industrial production process in its underlying technology. Using various low-power sensors and Internet of Things communication, the status of a manufacturing workshop's production line can be uploaded in real time to a big data analysis platform. Then, adjustments can be made to the operation speed of equipment, the preparation of materials, and the scheduling of workers in order to achieve the efficient connection and proximity of numerous production links. Cooperation will increase the efficiency of industrial production. R&D and design technologies such as laser scanning, holographic scanning, augmented reality, virtual reality, and digital simulation enable R&D personnel to understand the performance indicators of prototypes using industrial software alone, as opposed to examining the physical objects at each step. As stated previously, the preceding verification reduces research and development costs and accelerates the introduction of new products. According to the company, the widespread use of visual recognition technology and industrial robots for product quality control helps to overcome the shortcomings of inconsistent manual-quality inspection standards and low-quality inspection efficiency, resulting in stable and consistent product quality throughout the manufacturing process. As a result, digital technology can be implemented into industrial production processes relatively quickly. The application of digital technology to the service industry requires companies to first identify the pain points in the provision of products and services, then identify digital technologies that match the pain points, and finally communicate with professional and technical personnel to design a terminal product. Consequently, digital transformation may necessitate stricter conditions and a longer effective period for the growth of service industry organizations.

### 2.3. Influence

First and foremost, digital transformation contributes to the development of the value of data and the improvement of labor efficiency.

Due to the rapid growth of the digital economy, data have become a valuable asset for companies of all sizes. Data must be mined for its application value, intelligent operations must be implemented, and overall operation efficiency must be enhanced in order to achieve digital transformation. The Internet of Things has enabled communication between machines and the manufacturing process. The production automation system has the ability to coordinate in real time and is coupled with enterprise information management, allowing for collaborative product life cycle management based on data integration, which increases production efficiency. For decision support, we can adapt the structure of functional departments to change business requirements, achieve seamless integration of data flow and business process among various departments, and develop a data-driven decision-making system as well as a management and control system that enhances the decision-making ability and efficiency. Utilizing digital technology allows the enterprise's production and operation status, as well as market dynamic information, to be rapidly aggregated and distributed to the decision-making management team, thereby creating the conditions for the enterprise to implement rapid response in order to obtain or maintain a competitive advantage.

Digital transformation can, for the second time, result in more intelligent operations and lower operating expenses.

In terms of enterprise organization, digital transformation can aid businesses in developing flexible support departments, adapting the structure of functional departments in response to shifting business requirements and gradually transforming a static organization into a living organism. A flexible support department can not only help organizations respond quickly to shifting market trends, but it can also significantly reduce human expenses and prevent extreme situations such as when there are no projects, followed by no projects again. Create a platform for human resources, for instance, to facilitate the sharing of talents within the organization. As soon as possible during the incubation period of new projects, form a team of two to three individuals to serve as the project incubation team and clarify quantitative assessment objectives to maximize the use of internal talent and reduce labor costs. By utilizing digital technology, businesses can obtain real-time information regarding their production and warehouse operations. In addition, enterprises can construct a more flexible supply chain and reduce production costs by utilizing big data technology to anticipate market demand fluctuations. As an additional step in the R&D process, various product parameters are digitized, a digital twin model of the product is created, and debugging is performed on the virtual software platform to realize the R&D process based on digital simulation, which reduces the cost of trial and error and shortens the R&D cycle, resulting in a substantial reduction in R&D costs.

Thirdly, digital transformation has the potential to strengthen internal controls and reduce operational risks.

Internal control is a crucial operation and management measure for businesses, as it helps reduce the risk of violating laws and regulations, preserves the authenticity and integrity of financial information, and promotes the company's long-term growth. When it comes to performing the function of internal control, success depends not only on the attention of the enterprise's top management, the caliber of the internal control department's personnel, and the availability of resources but also on the sophistication of internal control management tools. In recent years, cloud computing, big data, and the Internet of Things have been the most prominent examples of organizations' increasing use of sophisticated technologies for internal control work. Internal control management can be integrated into the business process with the aid of digital transformation, allowing it to interact with and monitor the complexities of market strategy and company operations. The accurate and timely detection of unsuitable business processes will contribute to the resolution of long-standing, intractable problems in the internal control system and the business system, as well as the identification of actual risks in operation management and risk mitigation strategies. Historically, risk management in the enterprise's internal control system has been limited to implementing countermeasures after a risk has occurred. In recent years, the utilization of digital tools has transformed internal control risk management from a passive response to an active identification process. Moreover, dynamic analysis, forward-looking control, and early warning are employed to assist.

## 3. Financial Crisis Early Warning Model

This study uses data from the financial indicator analysis section and EVA, a special section of the CSMAR database, to select financial early warning indicators. Based on the findings of previous research, Table 1 outlines the seven first-level financial indicators selected for analysis.

Since the index data collected has a wide range of dimensions and a large amount of data, the PCA method is used for dimensionality reduction preprocessing before inputting the original data into the CNN model for training. This allows us to screen out more accurate indicators before training the CNN model. The maximum and minimum values are then normalized in order to reduce the influence of different dimensions on the results and to improve the resilience and efficacy of the model as a consequence.

The data matrix is as follows:

(1) $$\begin{array}{c} X=\left[\begin{array}{cccc} X_{11} & X_{12} & \cdots & X_{1n}\\ X_{21} & X_{22} & \cdots & X_{2n}\\ \vdots & \vdots & \vdots & \vdots \\ X_{m1} & X_{m2} & \cdots & X_{mn} \end{array}\right]. \end{array}$$

The zero-average operation is as follows:

(2) $$\begin{array}{c} {\hat{X}}_{ij}=x_{ij}-\mu \left(x_{j}\right). \end{array}$$

The covariance matrix is as follows:

(3) $$\begin{array}{c} D=\frac{1}{m-1}{\hat{X}}^{T}\hat{X}. \end{array}$$

The *k*-dimensional data after dimensionality reduction are as follows:

(4) $$\begin{array}{c} Y=p\hat{X}. \end{array}$$

On this basis, the data in the matrix *Y* are normalized, and the processing method is as follows (5):

(5) $$\begin{array}{c} Y_{ij}^{\prime }=\frac{Y_{ij}-\min\left\{Y_{j}\right\}}{\max\left\{Y_{j}\right\}-\min\left\{Y_{j}\right\}}. \end{array}$$

After zero-averaging the training data, the principal component analysis is performed to minimize the input parameters of the convolutional neural network model and the correlation between input components. Afterwards, a convolutional neural network model is trained using the new sample data set obtained via principal component analysis. Gradient descent is used to continuously adjust the parameters of the convolutional neural network in order to obtain the final convolutional neural network model. Applying test sample data to the model to determine its predictive accuracy is the final step.

The calculation formula of the convolutional layer is as follows:

(6) $$\begin{array}{c} X^{\left(l\right)}=f\left(W^{l}⊗X^{\left(l-1\right)}+b^{\left(l\right)}\right). \end{array}$$

The output of bias *b* is as follows:

(7) $$\begin{array}{c} \max\left(0,\omega x+b\right). \end{array}$$

The cross-entropy cost is as follows:

(8) $$\begin{array}{c} y_{j}\ln\;a_{j}^{L}+\left(1-y_{j}\right)\ln\left(1-y_{j}\right). \end{array}$$

The weighted sum of squares is as follows:

(9) $$\begin{array}{c} \sum \omega^{2}. \end{array}$$

The final loss of adding *L*_2_ is as follows:

(10) $$\begin{array}{c} C=-\frac{1}{n}\sum \left[y_{j}\ln\;a_{j}^{L}+\left(1-y_{j}\right)\ln\left(1-y_{j}\right)\right]+\frac{\lambda }{2n}\sum \omega^{2}. \end{array}$$

## 4. Results

This research includes a sample of 200 companies in China's Shanghai and Shenzhen A-share nonfinancial sector listed companies whose net profit is less than zero for two consecutive years from 2014 to 2021 and implemented ST, as well as a sample of 1,000 financially sound companies, for a total of 1200 enterprise data. When PCA is used to achieve dimensionality reduction, the dimension value after dimensionality reduction is set to 30. The CNN model's hyperparameters are set according to the previous experience and grid search. The CNN learning function is represented by Adam, a university student. 0.001 is the rate, 0.5 is the abandonment ratio, the cost function is the cross-inheritance loss function, 64 is the size of each training batch, 10 is the learning duration, and 25% of the total data comes from the test data set. The ratio of the training set and test set is 8 : 2. In order to ensure the fairness of the comparison, the parameter settings in this paper are consistent with those in [7, 12].

**Figure 1:**
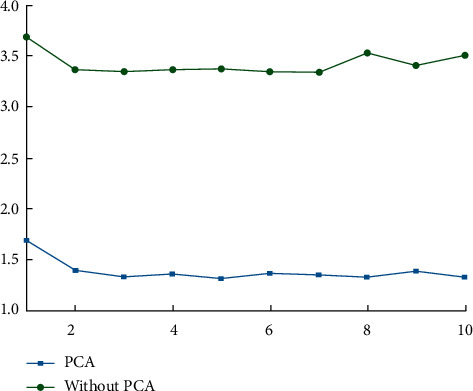
Comparison of training time.

**Figure 2:**
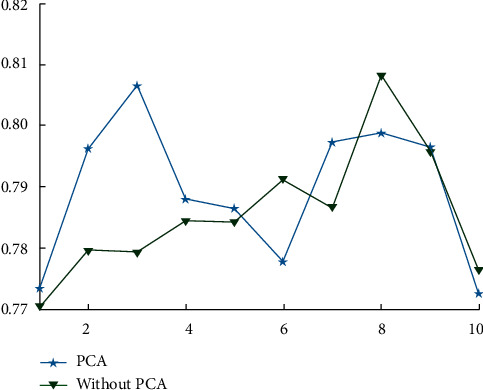
Comparison of accuracy.

First, it is established that the PCA method must be used to screen out the major components that have a significant impact on FCEW before further analysis can be carried out. The influence of PCA and non-PCA on the training duration and prediction accuracy of the developed CNN model is depicted in Figures 1 and 2, respectively. The training rate of the model can be significantly improved by using the PCA method after the interference index information has been removed, as can be shown from Figure 1.  Figure 2 illustrates that despite the fact that the PCA approach reduces the number of components needed for model training, it is capable of retaining the same amount of information as the original data. Accuracy is comparable. This is due to the fact that the PCA approach is used to extract the effective orthogonal eigenvalues from the original data, which are capable of summarizing the information contained in the original data to the greatest extent possible, as previously stated.

**Figure 3:**
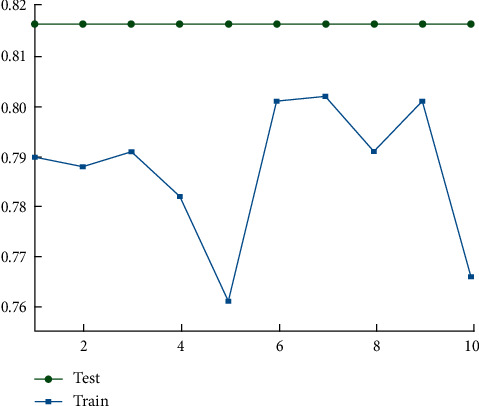
Accuracy comparison between the test set and the training set.

To determine the efficacy of model training, test data were fed into a previously trained model, and the accuracy of the test data was measured. Using the training set as an example, as depicted in Figure 3, the model's accuracy on the training set was 77.61 percent, and on the test set, it was 82.33 percent, indicating a more satisfactory training result. This result demonstrates that the accuracy of the test set is only marginally superior to the accuracy of the training set, indicating that the model is highly generalizable.

**Figure 4:**
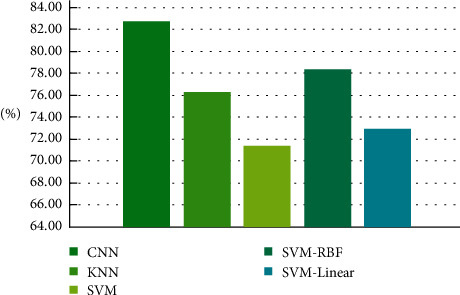
Comparison of accuracy of different models.

Specifically, this study compared the CNN-based FCEW model to the standard classic machine learning early warning approaches in order to demonstrate the usefulness and superiority of the CNN-based FCEW model. The following approaches were utilized for comparison: KNN, SVM, SVM-RBF, and SVM-linear. The basic parameter settings for this experiment are the same as those for the previous experiment, and the comparison findings are provided in Figure 4. On the basis of the results of this investigation, it can be concluded that the FCEW model developed in this study has a prediction accuracy of 82.67%, demonstrating a good financial forecast effect that is superior to other machine learning early warning methods. Due to the fact that the CNN can more effectively understand the association between multiple indicators and extract the most useful abstract characteristics, the accuracy of the prediction results may be ensured by using the CNN.

## 5. Conclusion

It is a revolution, a battlefield in the new global competitive landscape, and a long-distance race for which all employees must be prepared, and businesses must, among other things, actively embrace the various changes brought about by digitalization. This paper examines the heterogeneity of digital transformation's impact on enterprise growth, the mechanism of digital transformation's impact on enterprise growth, and the relationship between digital transformation and enterprise growth. Based on the CNN data, the study also develops a financial early warning model that will be used to guide the corporation through its digital transformation process.

## Figures and Tables

**Table 1:** FCEW symbols.

Class	Symbol	Definition
Profitability	*X*_1_	Net profit
Solvency	*X*_2_	Current assets
Growth ability	*X*_3_	(Total assets at year-end-total assets at the early year)/total assets at early year
Operating ability	*X*_4_	Main business income/total assets
Cash flow	*X*_5_	Liquidity
EVA	*X*_6_	Return on invested capital
Risk level	*X*_7_	Leverage

## Data Availability

The data used to support the findings of this study are available from the corresponding author upon request.
